# Gene polymorphisms of TNF-238G/A, TNF-308G/A, IL10-1082G/A, TNFAIP3, and MC4R and comorbidity occurrence in a Romanian population with psoriasis

**Published:** 2018

**Authors:** Vlad Mihai Voiculescu, Iulia Solomon, Alexandra Popa, Carmen Cristina Draghici, Maria Dobre, Calin Giurcaneanu, Laura Maria Lucia Papagheorghe, Mihai Lupu

**Affiliations:** * Discipline of Clinical Dermatology and Allergology, „Carol Davila” University of Medicine and Pharmacy, Bucharest; ** Dermatology Department, „Elias” University Emergency Hospital, Bucharest; *** „Victor Babeș” National Research Institute, Bucharest; **** Dermatology Ambulatory Center, „Colțea” Clinical Hospital, Bucharest; ***** Department of Dermatology, MEDAS Medical Center, Bucharest

**Keywords:** Metabolic Syndrome, Polymorphism, Single Nucleotide, Psoriasis, Comorbidity, Cytokines

## Abstract

**Rationale.**Psoriasis is a prevalent chronic inflammatory disease with worldwide distribution affecting approximately 2% of the Caucasian population. There have been many population- and family-based studies that agree on the strong genetic component of this disease. Several studies have investigated the relationship between cytokine gene polymorphisms, psoriasis, and the occurrence of comorbidities but their data are conflicting.

**Objective.**This study examines cytokine gene single-nucleotide polymorphisms (SNPs) in the context of psoriasis and metabolic syndrome, with a focus on the occurrence of comorbidities in psoriasis patients. The working hypothesis is that particular SNPs may predispose to an accelerated disease course and more comorbidities in psoriasis patients.

**Methods:**This cross-sectional study was carried out in 2016 in the Dermatology Department of “Elias” University Emergency Hospital, Bucharest and included 82 psoriasis patients. Several clinical and laboratory parameters were recorded, and the presence of metabolic syndrome (MetS) was noted. Using real-time PCR, we tested for the following SNPs: rs361525, rs1800629, rs1800896, rs610604, rs17782313.

**Results:**Disease severity was not significantly influenced by any of the five studied SNPs. Gene polymorphism of rs17782313 was found to influence the occurrence of psoriatic arthritis. In these patients, rs610604 and rs17782313 polymorphisms were associated with the presence of diabetes mellitus. Furthermore, rs17782313 influenced the presence of obesity, heterozygotes being more at risk. Our data suggested that MetS occurred independently of the five studied SNPs.

**Discussion.**The influence of certain cytokine gene polymorphisms on multiple organ systems is justification enough for further analysis of the genetic and molecular mechanisms of metabolic syndrome development in psoriasis patients.

**Abbreviations:**single-nucleotide polymorphisms – SNPs, metabolic syndrome – MetS

## Introduction

Psoriasis is one of the most frequently encountered and yet one of the least understood inflammatory skin diseases in current clinical practice. It is a chronic inflammatory disease that is more prevalent in Caucasians (approximated at 1.5-3%) with a prevalence peak between 3-4.8% in Norway [**1**][**2**][**3**].

The progress made by genetic studies in the last decades [**3**] and the efforts that have been put into understanding its immunological mechanisms have given birth to the current concept that psoriasis is a systemic inflammatory disease with a strong genetic component [**4**].

The permanent subclinical inflammatory state in psoriasis patients seemingly leads to an increased cardio-cerebro-vascular morbidity in these patients, when compared to the general population. Several studies have associated severe psoriasis with increased cardiovascular morbidity and mortality [**5**][**6**][**7**][**8**]. This could be explained through the abnormal lipid metabolism [**5**][**9**][**10**][**11**][**12**][**13**] as well as the higher rate of comorbidities such as diabetes mellitus (DM), arterial hypertension and obesity, often seen in psoriasis patients [**5**][**6**][**14**][**15**][**16**]. Even though a Finnish study has reported increased mortality rates in psoriasis patients [**17**], some authors suggest there is no definitive evidence that having a chronic plaque or pustular psoriasis alone reduces an individual’s lifespan [**3**].

Metabolic syndrome (MetS) is defined as the association between insulin resistance, systemic inflammation, central type obesity, atherogenic dyslipidemia, and arterial hypertension. Metabolic syndrome has been proven to represent a multi-system aggressor, affecting the musculoskeletal, digestive, endocrine and cutaneous systems. In their meta-analysis, Armstrong AW et al. found a higher prevalence of MetS in psoriasis patients when compared to the general population and a direct proportionality relation between psoriasis severity and the risk of developing MetS [**18**].

The role of TNF-α in psoriasis immunopathological processes has already been established. This proinflammatory cytokine was found to have increased serum levels as well as increased in situ levels in psoriastic skin lesions.

Single nucleotide polymorphisms (SNPs) are the most common type of genetic variation. An SNP represents a mutation characterized by the difference of a single nucleotide in the DNA of a gene. Some SNPs can influence the affected gene’s activity.

This study is aimed towards proving the association between the presence of particular SNPs in psoriasis patients and evidence of accelerated disease progression, higher disease severity, and/or a higher number of comorbidities.

## Materials and methods

This cross-sectional study was carried out between April 2014 and May 2016. The patients were consecutively enrolled in the study upon being clinically diagnosed with psoriasis in the Dermatology Department of the ‘ELIAS’ University Emergency Hospital in Bucharest. Inclusion criteria were defined as having a clinical diagnosis of psoriasis, age between 18 and 90 years old, whereas patients with systemic infectious or neoplastic diseases were excluded from the study. After meeting the inclusion criteria, all patients had given informed consent, the study being carried out in conformity with the World’s Medical Association Declaration of Helsinki and national norms (law no.46/2003 on patients’ rights).

Several clinical and laboratory parameters were collected in charts designed especially for the study, including a unique identification number assigned to each patient, age, sex, height, weight, PASI (Psoriasis Area and Severity Index) score (range 0-72) [**19**], DLQI (Dermatology Quality of Life Index) score, MetS criteria (as defined below), common blood analysis data, and comorbidity data.

Psoriasis disease severity was established as mild (PASI ≤10) and moderate to severe (PASI >10) as per the European consensus (2011) [**20**].

Metabolic syndrome was defined using the 2006 IDF (International Diabetes Federation) criteria: central type obesity (abdominal circumference ≥94 cm in men and ≥80 cm in women) and any two of the following: fasting blood glucose level over 100 mg/dl or an existing diagnosis of DM, serum HDL cholesterol levels <40 mg/dl in men and <50 mg/dl in women, serum triglyceride levels >150 mg/dl, blood pressure values over 130/85 mmHg or existing antihypertensive treatment [**21**].

After meeting the inclusion criteria and having signed an informed consent form, biological samples from patients were obtained in the Dermatology Department of the ‘ELIAS’ University Emergency Hospital in Bucharest. Complete blood count analysis was performed using the Sysmex XT 4000i automatic system. Blood biochemistry was performed on the automated Vitros 350 systems with Johnson&Johnson reagents using the dry chemistry method and Architect c8000 systems by wet chemistry methods. CRP immunology was done by latex-agglutination methods and immunoturbidimetry on the Immulite 2000 system.

Genotyping tests were performed in the „Molimagex” Molecular Biology Research Laboratory of the Emergency University Hospital of Bucharest. Peripheral venous blood was collected in the dermatology ward of Elias University Emergency Hospital, after trial inclusion of the patients. Each vacuum tube (Vacutainer, BD Biosciences) received an alphanumeric code, followed by the insertion of this code in a database. The anticoagulant-treated tubes were kept refrigerated (at -28°C) until all the needed material was collected. The DNA necessary for genotyping was extracted and quantified using the QIAamp DNA Mini Kit (QIAGEN), after a time interval between 2 and 6 months after sample collection. We opted for extraction from the leukocyte band which was obtained by centrifugation because a higher quantity of DNA can be obtained in this way from the same volume of a 200 µl sample. The quantification of extracted DNA was done by computing the concentration of the DNA obtained through the washing process, by measuring the absorption rate at 260 nm wavelength using a Tecan infinite 200pro spectrophotometer and by fluorometric analysis using an Invitrogen Qubit fluorometer. The purity was determined by computing the absorption ratio at 260 nm and 280 nm.

After having verified the quantity and purity of the extracted DNA, we proceeded to the genotyping phase by means of real-time PCR (rt-PCR) using TaqMan® (Life Technologies, USA) probes, specific to each studied allele, coupled with 2 different fluorochromes.

In light of the data from previous studies, the following SNPs were studied:

• rs361525 also known as TNF-238 G/A, located on chromosome 6, TNF gene, position 31575324 in the promoter region; • rs1800629 also known as TNF-308 G/A, located on chromosome 6, TNF gene, position 31575254 in the promoter region; • rs1800896 also known as IL10-1082 G/A, located on chromosome 1, position 206773552; • rs610604, located on chromosome 6, TNFAIP3 gene, position 137878280; • rs17782313, located on chromosome 18, MC4R gene, position 60183864.

**Data analysis.**Raw data were collected using the Light Cycler® 480 Gene Scanning Software and then imported into the LightCycler® 480 Endpoint Genotyping system which determined the genotype of a sample based on its fluorescence emission in one of the following: ancestral homozygote (AA) or mutant (MM) and heterozygote (AM). Statistical analysis was performed using SPSS v.20 (IBM, USA) with significance being established at ≤0.05 (CI 95%). Student’s T-test, Chi-square, and Mann-Whitney U tests were used for testing the differences between groups, in accordance with the type of data being analyzed (normal or non-normal distributions, continuous or categorical data).

## Results

In total, 82 psoriasis patients were enrolled in the study: 46 males and 36 females with a mean age of 49.96 ± 14.54 years. The median disease duration was 14.64 years with a mean age at diagnosis of 36.31 ± 15.85 years. Thirty-seven patients fulfilled the aforementioned MetS criteria, whereas 32 did not and for 13 patients sufficient data could not be collected.

There was no statistically significant difference in disease severity between the group associating psoriasis and MetS and the group with psoriasis alone at enrollment (p= 0.225).

Patients presenting both psoriasis and MetS were, on average, 11.56 years older than patients with psoriasis alone (t= -3.957, p<0.001, d= 0.91). Also, subjects associating MetS were diagnosed with psoriasis 8.38 years later, on average (t= -2.4, p= 0.019, d= 0.554) (**Table 1**).

**Table 1:** Clinical characteristics of the study groups

	Psoriasis alone	Psoriasis and MetS	p value
Number of patients (N*)	43	39	N/A
Men	22	24	N/A
Women	21	15	N/A
Mean age ± SD† (years)	44.47 ± 15.55	56.03 ± 10.57	p<0.001
Mean age at diagnosis ± SD (years)	32.23 ± 14.86	40.61 ± 15.91	p= 0.019
Psoriasis artropathy (N)	3	10	p= 0.023
PASI‡ (median)	6.8	9.65	p= 0.225
*N= number; †SD= standard deviation; ‡PASI= Psoriasis Area and Severity Index; §MetS= metabolic syndrome			

**Disease severity.**Out of the 82 patients in the study group, more patients had mild disease severity (N=42), whereas in 9 patients disease severity could not be established due to missing data. Also, the moderate to severely affected patients fulfilled significantly more (p= 0.01) MetS criteria when compared to the mild severity group. Data analysis did not uncover any influence of the five studied SNPs on disease severity in our patients: rs361525 (p> 0.05), rs1800629 (p> 0.05), rs610604 (p= 0.601), rs1800896 (p= 0.125), and rs17782313 (p= 0.440).

**Fig. 1:**
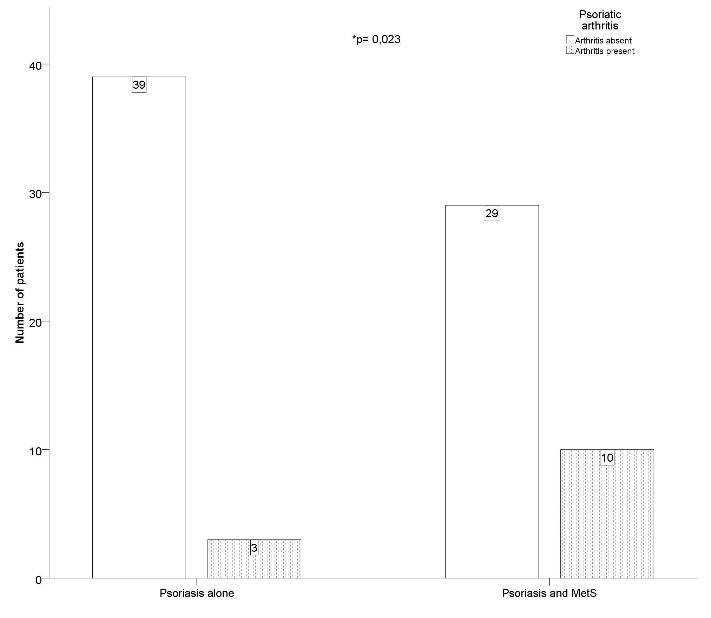
Significantly more patients were affected by psoriatic arthritis when metabolic syndrome was present

**Psoriatic arthritis and metabolic syndrome.**The number of patients showing PsA was significantly higher (χ2= 5.13, p= 0.023, V= 0.251) in those associating MetS compared to psoriasis alone patients (**Figure 1**). Moreover, the relative risk for developing PsA in patients with both psoriasis and MetS was 3.58 times higher than that of patients with psoriasis alone.

SNPs influence psoriatic arthritis. Our data suggest that SNP rs17782313 had a significant influence on the development of PsA (χ2= 5.374, p= 0.05, V= 0.271). Significantly more of the TT genotype patients (N= 8) were affected by arthritis than CT (N= 2) or CC (N= 2) patients. The remaining four SNPs did not show any influence on PsA in our statistical model.

**Fig. 2:**
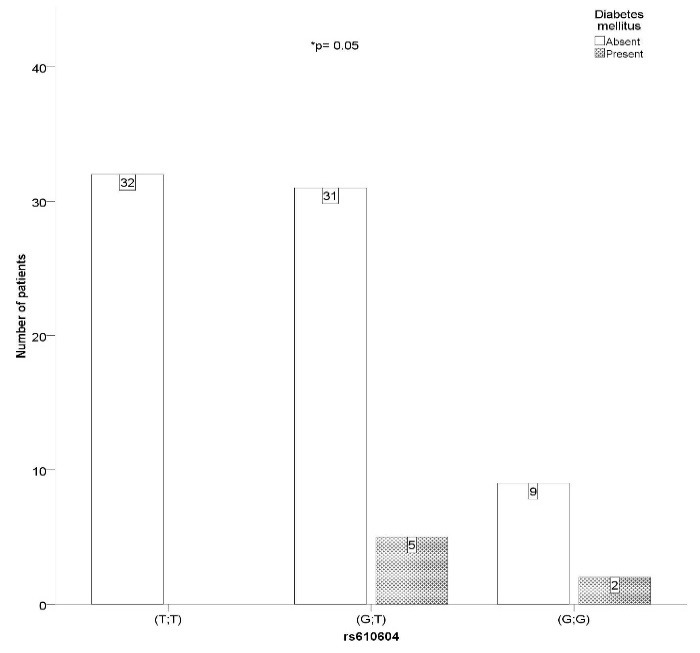
SNP rs610604 GT genotype is associated with the presence of diabetes mellitus

**Linking SNPs, diabetes mellitus, obesity and MetS.**We have found SNP rs610604 to be significantly associated with the presence of DM in our study group (χ2= 5.422, p= 0.05, V= 0.265). Significantly more rs610604 GT genotypes (N= 5) also met the DM criteria compared to the GG genotype patients (N= 2). Also worth mentioning is that no diabetic patients were found among the TT genotypes (**Figure 2**).

**Fig. 3:**
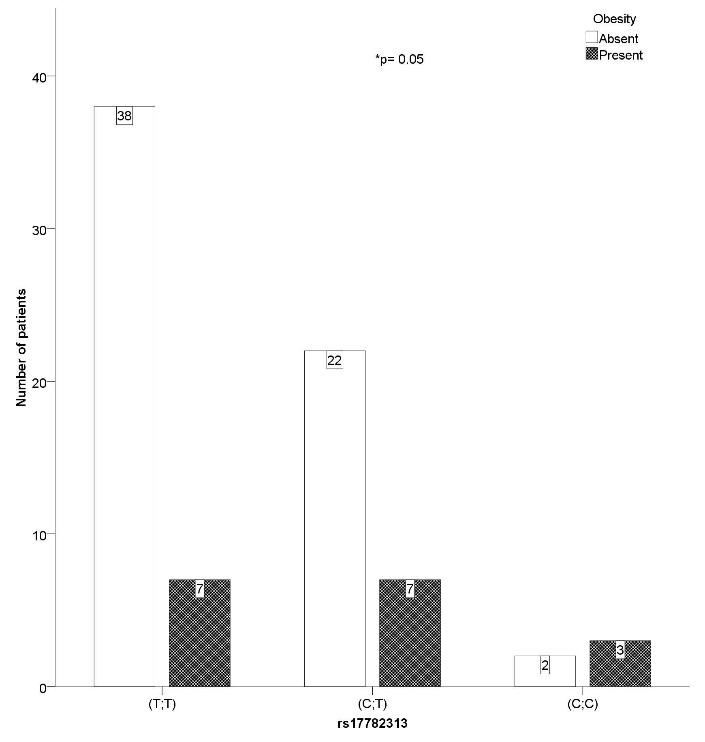
SNP rs17782313 CT genotype patients have a relative risk of 2.33 for developing obesity compared to CC genotypes

Our data suggests that SNP rs17782313 also played a significant part in the presence of diabetes (χ2= 6.525, p= 0.038, V= 0.298). A higher number of CT genotypes met the DM criteria compared to the TT homozygotes (5 vs. 2). Furthermore, SNP rs17782313 significantly influenced the presence of obesity (χ2= 5.449, p= 0.05, V= 0.273), with CT heterozygotes having a relative risk for obesity of 2.33 compared to the CC genotype patients (**Figure 3**).

**Fig. 4:**
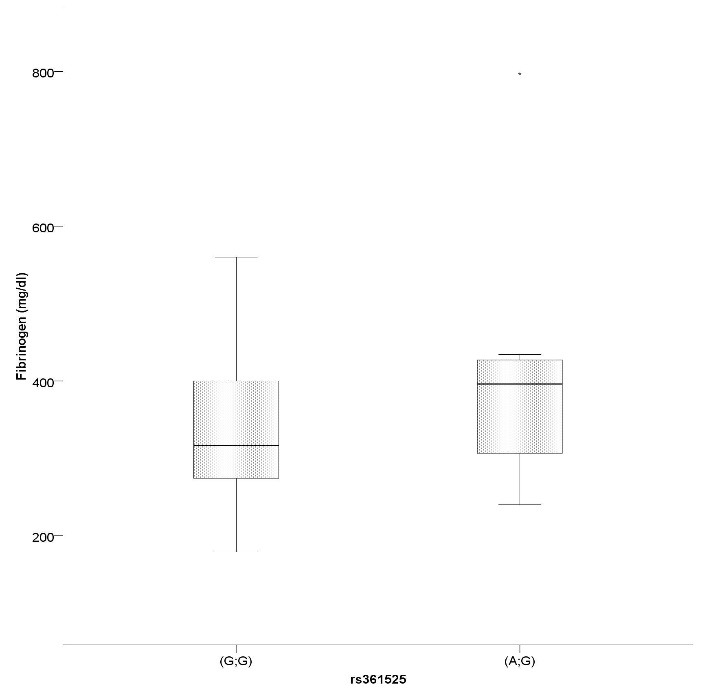
SNP rs361525 AG heterozygotes have significantly higher serum fibrinogen levels than GG genotypes

Biological variables such as erythrocyte sedimentation rate, cholesterol, triglycerides and serum fasting glucose did not appear to be influenced by any of the studied polymorphisms. However, we did find a significant mean difference of 71.771 mg/dl in serum fibrinogen levels between rs361525 AG and GG genotypes (t= 1.797, p= 0.05, d= 0.184) (**Figure 4**).

Finally, the metabolic syndrome appeared to be independent of all five SNPs studied in our patients.

## Discussion

Psoriasis patients have been found, by multiple studies, to have an increased risk of diabetes mellitus [**22**][**23**]. Central type obesity plays a key role in MetS development and tends to precede the occurrence of all its other components [**24**]. Several studies have also identified a chronic low-level inflammatory state in obese individuals [**25**].

Stimulated by activated macrophages in adipose tissue, adipocytes secrete adipokines such as IL-6, TNF-α, visfatin and leptin, which also play an important role in the pathogenesis of psoriasis [**26**][**27**][**28**]. The greatest body of supporting evidence refers to adiponectin and leptin [**25**]. Studies show that while circulating leptin levels correlate with fat mass and increased production of pro-inflammatory cytokines (TNF-α, and IL-6) [25], leptin also stimulates keratinocyte proliferation and angiogenesis [**29**]. High circulating leptin levels have been found in psoriasis patients [**14**][**30**] possibly deriving both from adipose tissue in obese patients and inflammation [**31**].

We have found that rs610604 and rs17782313 polymorphism is strongly correlated with the development of DM, an important and severe comorbidity in psoriasis patients, while the latter also correlates with the presence of obesity alone. The rs17782313 (C) allele has been significantly associated with higher intakes of dietary fat, the SNP being related to greater long-term weight change and an increased risk of obesity and diabetes [**32**], especially in women [**33**]. A study conducted on 60,000 adults indicates an association between rs17782313 (C) alleles and a higher body mass index (BMI), with an average increase of 0.22 BMI units (p = 2.8x10-15) [**34**].In our study, SNP rs17782313 has been found to favor the development of psoriatic arthritis.

Our data confirm that patients associating psoriasis and MetS do indeed appear to have a higher risk of developing psoriatic arthritis, a severe and debilitating complication.

The most studied TNF-α polymorphism is by far the substitution of guanine (G) with adenine (A) in the -308 position of the promoter region, also known as rs1800629. The presence of adenine (A allele) in this position has been previously associated with increased TNF-α when compared to the G allele [**35**].

Rs361525 polymorphism, also known as TNF-238, is a SNP in the TNF-α gene which has been linked to a wide variety of conditions: breast cancer [**36**], Graft-versus-host disease [**37**], psoriasis, diabetes, glaucoma [**38**], lymphoma [**39**] and many others. A Polish study conducted on 160 psoriasis patients reported an increased rs361525(A) allele frequency (16.8% vs. 3.1%, p=0.000017, OR= 8.79, CI: 2.606-29.678). Moreover, the onset age was lower in rs361525(A) carriers [**40**]. In our study, there was no difference in age at diagnosis between the rs361525(A) and rs361525(G) patients (36.22 vs. 36.32 years old, p= 0.986). Yu GI et al. analyzed rs361525 in 123 control and 208 overweight/obese subjects in a Korean population. The results of this study suggest that the G allele of rs361525 in the TNF-α gene may be a risk factor for overweight/obesity in the Korean population, with the frequency of the G/G genotype in the overweight/obese group being 9.3% higher than that in the control group (p= 0.0046) [**41**].

A rather peculiar result, against all expectations, was the lack of influence in the statistical model of these particular SNPs, rs361525 and rs1800629. This could very well be influenced by the low frequency of some genotypes in the general population, coupled with the relatively low sample size.

The present study only included patients from one dermatology department, thus the results cannot be in any way extrapolated. The study was also limited by the small sample size which might have affected the statistical power, the median observed study power being 40%. Even so, the Hardy-Weinberg equilibrium was kept, thus refuting any selection pressures on the studied population. However, much larger, prospective studies, are needed in order to firmly establish the influence of certain SNPs in the development of comorbidities in psoriasis patients.

Collectively, our results obtained in a Romanian population expand current knowledge regarding the link between certain polymorphisms in psoriasis patients and a higher number of comorbidities, as well as psoriatic arthritis.

Even though these polymorphisms were studied in relation to psoriasis, it is very likely they could determine phenotypic expressions with echoes in other organ systems, which are yet to be uncovered. These associations pave the way for more complex studies investigating the genetic and molecular mechanisms of the development of metabolic syndrome in psoriasis patients. Now, more than ever, dermatologists should play a key role in identifying at-risk patients, and implementing prophylactic and management strategies. Tailored genotyping and individualized treatment represent, in our view, future directions towards a more efficient management of psoriasis patients.

**Acknowledgment**

We would like to thank Mrs. Gina Manda for her guidance and support with scientific materials.

**Source of funding:**

This article was partly funded by the “Young Researchers” Grant from UMF “Carol Davila” Bucharest, number 33880/11.11.2014.

**Disclosures:**

The authors declare that there is no conflict of interest.
